# Near infrared bioimaging and biosensing with semiconductor and rare-earth nanoparticles: recent developments in multifunctional nanomaterials

**DOI:** 10.1039/d1na00502b

**Published:** 2021-10-06

**Authors:** Artiom Skripka, Diego Mendez-Gonzalez, Riccardo Marin, Erving Ximendes, Blanca del Rosal, Daniel Jaque, Paloma Rodríguez-Sevilla

**Affiliations:** Nanomaterials for Bioimaging Group, Departamento de Física de Materiales, Facultad de Ciencias, Universidad Autónoma de Madrid Madrid 28049 Spain paloma.rodriguez@uam.es daniel.jaque@uam.es; The Molecular Foundry, Lawrence Berkeley National Laboratory Berkeley California 94720 USA; Instituto Ramón y Cajal de Investigación Sanitaria (IRYCIS) Ctra. Colmenar km. 9.100 Madrid 28034 Spain; ARC Centre of Excellence for Nanoscale BioPhotonics, School of Science, RMIT University 124 La Trobe St Melbourne VIC 3000 Australia

## Abstract

Research in novel materials has been extremely active over the past few decades, wherein a major area of interest has been nanoparticles with special optical properties. These structures can overcome some of the intrinsic limitations of contrast agents routinely used in medical practice, while offering additional functionalities. Materials that absorb or scatter near infrared light, to which biological tissues are partially transparent, have attracted significant attention and demonstrated their potential in preclinical research. In this review, we provide an at-a-glance overview of the most recent developments in near infrared nanoparticles that could have far-reaching applications in the life sciences. We focus on materials that offer additional functionalities besides diagnosis based on optical contrast: multiple imaging modalities (multimodal imaging), sensing of physical and chemical cues (multivariate diagnosis), or therapeutic activity (theranostics). Besides presenting relevant case studies for each class of optically active materials, we discuss their design and safety considerations, detailing the potential hurdles that may complicate their clinical translation. While multifunctional nanomaterials have shown promise in preclinical research, the field is still in its infancy; there is plenty of room to maximize its impact in preclinical studies as well as to deliver it to the clinics.

## Abstract 2

This review highlights the very recent examples of near infrared contrast agents employed for multivariate diagnostics, multimodal imaging, and theranostic. Considerations on how to further advance these probes towards real-life use are also given.

## Graphical abstract

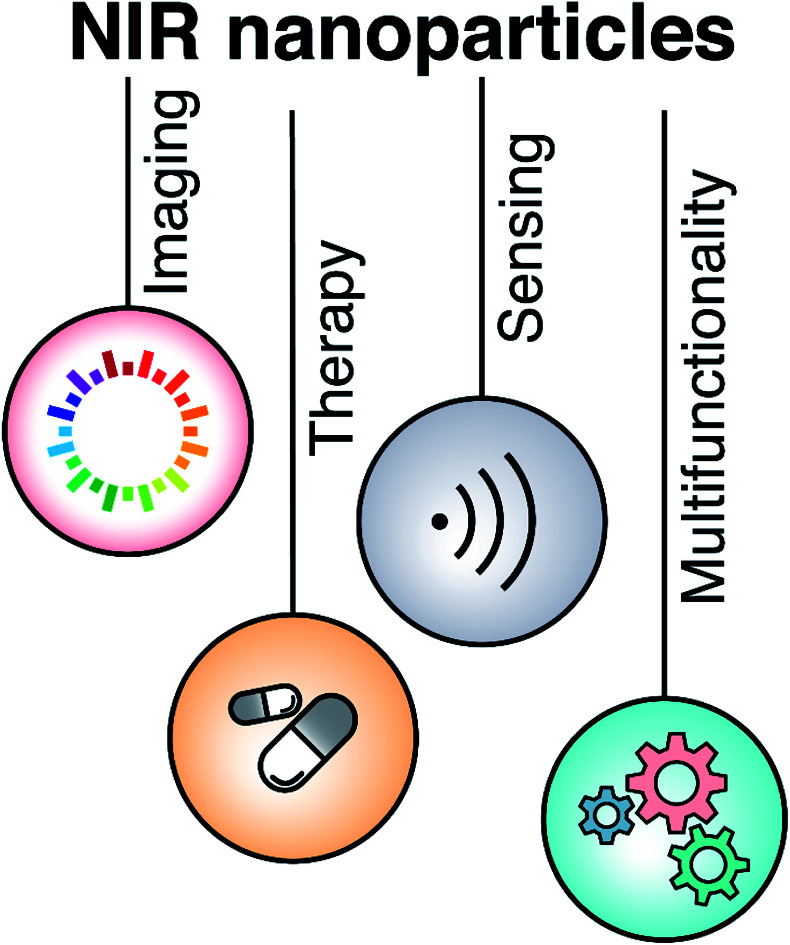

## 1. Introduction

Current clinical diagnostic methods take advantage of several mature imaging modalities – magnetic resonance imaging (MRI), X-ray computer tomography (CT), positron emission tomography (PET), ultrasonography, *etc.* – to deliver rapid and accurate disease detection. The strive for even faster, more cost-effective, and less invasive ways to address healthcare problems nonetheless continues, and is being developed at an unprecedented speed when it comes to optical tools.

**Fig. 1:**
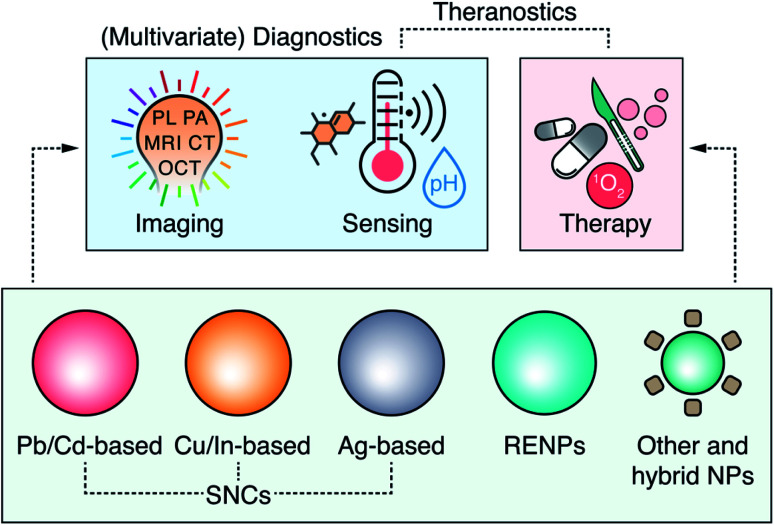
Overview of the major nanoparticle (NPs) types (including different semiconductor nanocrystals, SNCs, and rare-earth doped nanoparticles, RENPs) extensively researched for imaging, sensing and therapy in the NIR – pushing forward the concepts of multivariate diagnostics and theranostics of personalized medicine.

Electromagnetic radiation within the optical spectrum, from ultraviolet to near infrared (NIR), can be generated with inexpensive, compact, easy to operate sources (lasers or lamps), and can provide real-time imaging at the microscale. The success of optical diagnostics, however, largely depends on an appropriate selection of the irradiation wavelengths. NIR light (“light” here is used as a shorthand for radiation) interacts less with biological tissues than light of shorter wavelengths, and thus can propagate deeper within them. NIR wavelengths which experience reduced absorption and scattering by endogenous components of tissues cover two spectral regions – NIR-I (750–950 nm) and NIR-II (1000–1700 nm).^1–4^ While intrinsic tissue fluorescence in the NIR can provide diagnostic information in some specific scenarios, analogous to MRI or PET techniques, the potential of light-based imaging is maximized when paired with contrast agents, *i.e.*, markers that can absorb (or scatter) NIR wavelengths. The use of contrast agents enables NIR photoluminescence (PL),^5^ photoacoustic (PA),^6^ or optical coherence tomography (OCT) imaging.^7^ Nanotechnology has further allowed to design NIR contrast agents that accommodate multiple imaging modalities at once,^8,9^ enable multivariate diagnostics – combining image acquisition with qualitative or quantitative measurement of chemical or physical cues,^10–12^ and provide concurrent therapeutic possibilities (the principle of theranostics) (Fig. 1).^13–15^

NPs that act as multimodal contrast agents for two or more complementary imaging modalities remove the need for multiple contrast agents and allow achieving a more complete diagnostics picture. NPs capable of multivariate diagnostic could similarly reduce the number of diagnostic tests the patients would be subjected to. These NPs would provide clinicians with ample information (for example, presence of disease biomarkers or local chemical changes) that would facilitate accurate diagnosis. Beyond diagnostics, multifunctional theranostic NPs combine disease diagnosis and therapy. However, particular caution has to be taken with theranostic NPs to minimize damage to healthy tissues by timely activating the therapeutic function and limiting it to the diseased tissue.^16^ Despite the potential of multifunctional NPs to revolutionize clinical diagnosis and therapy, turning that into reality requires the development of the most suitable materials and fully ascertaining their effectiveness and safety for use in personalized medicine.

In this review, we discuss the most recent developments in multimodal imaging, multivariate diagnostics, and theranostics using optical NPs, providing the reader with an at-a-glance overview of the most promising materials studied for these applications (Table 1).

**Table 1:** Major types of NPs intended for biomedical applications (PL – photoluminescent, PA – photoacoustic, PTT – photothermal therapy), their primary characteristics, and clinical perspectives

Type	Select compositions	NIR emission range	Principal applications	Developmental maturity	Intrinsic toxicity	Current potential for clinical translation
Pb-based SNCs	PbS	NIR-I (>800 nm)	PL imaging and sensing	High	High	Low
		NIR-II (<1800 nm)				
Cd-based SNCs	CdSe, CdSeTe	NIR-I (<900 nm)	PL imaging and sensing	High	High	Low
Cu-based SNCs	CuS, Cu_2_S, CuInS_2_	NIR-I (>750 nm)	PL/PA imaging, sensing, and PTT	Medium	Medium	Medium
		NIR-II (<1300 nm)				
In-based SNCs	InP, InAs, InSb	NIR-I (>800 nm)	PL imaging	Low	Low	Medium
		NIR-II (<1600 nm)				
Ag-based SNCs	Ag_2_S, Ag_2_Se	NIR-II (1000–1600 nm)	PL imaging, sensing, and PTT	Low	Medium	Medium
RENPs	NaYF_4_, LiYF_4_	NIR-I (>800 nm)	PL imaging, sensing, and light-triggered therapy	High	Medium	Medium
		NIR-II (<1600 nm)				
Silicon NPs	Primarily Si	NIR-I (>750 nm)	PL imaging	Low	Low	High
		NIR-II (<1100 nm)				
Carbon-based	Primarily C	NIR-I (>750 nm)	PL imaging and PTT	Low	Low	Medium
		NIR-II (<1400 nm)				
Perovskite-like	LaAlO_3_, NaGdTO_3_	NIR-I (750–800 nm)	Persistent luminescence imaging	Low	Medium	Low

We focus on the four classes of materials that have attracted major research efforts over the past few years – Pb- and Cd-based semiconductor nanocrystals (SNCs), Cu- and In-based SNCs, Ag-based SNCs, and rare-earth doped nanoparticles (RENPs), with a final section focused on novel and hybrid systems. For each material, we present recent relevant case studies that we believe have significant implications for the future development of these tools targeted for personalized medicine. We also discuss the potential shortcomings that could limit the applicability of these NPs and outline the attempts that are being made to improve the existing materials, create novel ones, or form hybrid-structures that combine the strengths of their individual components. Finally, we propose a set of practices to be considered when designing, testing, and applying NIR-activated multifunctional NPs, highlighting the need for two-way communication between the professionals of physical and medical sciences.

## 2. Pb- and Cd-based SNCs

SNCs based on lead and cadmium are found in element groups II-VI (CdS, CdTe, CdSe) and IV-VI (PbS, PbTe, PbSe). This type of SNCs fulfil multiple requirements imposed over luminescent markers to be used for bioimaging, including large extinction coefficients spanning broad spectral ranges, narrow emission lines with high degree of spectral tunability, stability against photobleaching, and even near-unity PL quantum yields (PLQY).^17,18^ Lead chalcogenide SNCs are particularly relevant for NIR imaging, as their PL emission peak can be easily tuned to span wavelengths from 800 to 1800 nm coinciding with NIR-I or NIR-II spectral ranges.^19^

The distinctive qualifier of these SNCs is the presence of heavy metal ions (Pb^2+^, Cd^2+^) in their composition, which confers their unique spectral characteristics, while intrinsic toxicity raises well-warranted concerns about the safety and potential clinical translation of these NPs.^20^ Nonetheless, this is less of a concern for *in vitro* and some preclinical studies, where Pb- and Cd-based SCNs can excel. Research into deep-tissue PL imaging,^21^ targeted NP delivery and pharmacokinetics,^22^ and real-time monitoring of physiological events,^23^ have relied on these SNCs. The performance of other types of NPs is also often benchmarked against that of heavy-metal SNCs. Besides, colloidal synthesis methods employed in the preparation of Pb- and Cd-based SCNs have evolved to accommodate SNCs of various compositions, shapes, and sizes, down to a monolayer precision.^24^ In turn, this facilitates both the versatility and the reproducibility in SNCs manufacturing, which guarantees more predictable behavior in model biological organisms.

**Fig. 2:**
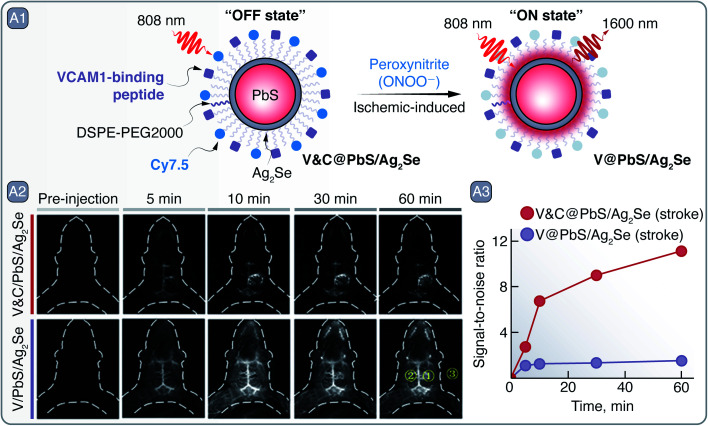
A – Ischemic stroke detection: A1 – schematic representation of V&C@PbS/Ag_2_Se SNCs and of their PL change in the presence of ONOO^−^. A2 – PL imaging in the NIR-II of an early ischemic stroke with V&C@PbS/Ag_2_Se and V@PbS/Ag_2_Se SNCs over a period of an hour after administration. A3 – quantitative assessment of signal-to-noise ratio change from the PL images. Mean intensities from the yellow regions of interest (ROI) in A2 were used to calculate the signal-to-noise ratio = (ROI1-ROI3)/(ROI2-ROI3). Adapted from ref. 25 with permission from John Wiley and Sons, copyright 2020 (CC BY 3.0).

As a first line of defense against heavy metal ion leaching, Cd- and Pb-based SNCs are often coated with heavy-metal-free inorganic shells and organic layers that additionally provide dispersibility in an aqueous environment. Yang *et al.* have prepared PbS/Ag_2_Se SNCs, coated with functionally modified phospholipids, which were devised as multivariate diagnostic markers of ischemic stroke regions (Fig. 2 – A).^25^ Structural stability in phosphate-buffered saline (PBS at pH 7.4) experiments showed that the Ag_2_Se shell over the PbS core of SNCs prevented Pb^2+^ leaching into the environment, poisoning the solution and decomposing the SNCs, as was the case for core-only PbS SNCs. The Ag_2_Se shell also shifted the PL of SNCs from ∼1586 nm for core-only PbS to around 1616 nm for PbS/Ag_2_Se. To selectively image ischemic stroke regions, these SNCs were functionalized so that their emission was turned on in the presence of peroxynitrite (ONOO^−^), a marker of early onset of an ischemic stroke. The PbS/Ag_2_Se SNCs were coated with phospholipids functionalized with VHPKQHR peptide and Cy7.5 organic dye (V&C@PbS/Ag_2_Se; Fig. 2 – A1). The peptide served as a guided delivery tag against VCAM1 protein expressed on the inflamed endothelial cells in the ischemic stroke regions and facilitated the uptake of the SNCs into the cells. The Cy7.5 dye quenched the PbS/Ag_2_Se SNCs emission by 89%, yet in the presence of ONOO^−^ the dye was oxidized and PL of the SCNs was restored (Fig. 2 – A1). The multivariate diagnosis capabilities of V&C@PbS/Ag_2_Se SNCs were tested on a photothrombotic stroke mouse model. After administration, V&C@PbS/Ag_2_Se SNCs were internalized by inflamed endothelial cells and their NIR PL signal could be observed due to Cy7.5 oxidation by ONOO^−^. The PL of V&C@PbS/Ag_2_Se SCNs was restored gradually over 60 min, clearly lighting-up the infarcted region (Fig. 2 – A2). In contrast, PbS/Ag_2_Se SNCs functionalized only with the targeting peptide (V@PbS/Ag_2_Se) illuminated the entire brain, with no apparent delineation between healthy and injured tissue (Fig. 2 – A2–3).

**Fig. 3:**
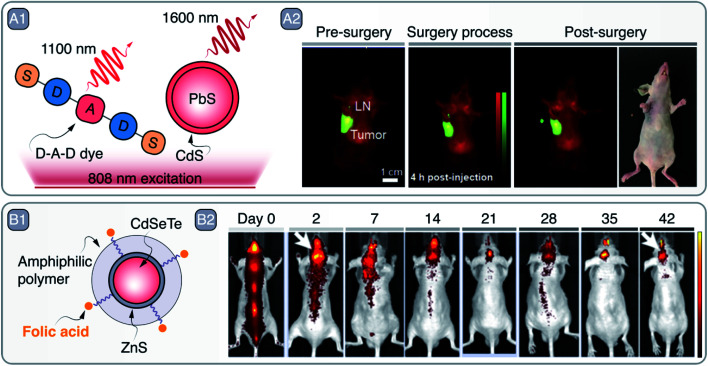
A – Sentinel lymph node (LN) imaging and excision: A1 – depiction of D–A–D dye and PbS/CdS SNCs used to image tumors and sentinel LNs under NIR excitation, respectively. A2 – PL imaging aided tumor and LN visualization pre-, during, and post-surgery; excised LN is shown in NIR as well as optical image. Adapted from ref. 30 with permission from John Wiley and Sons, copyright 2020 (CC BY 3.0). B – targeted glioma imaging: B1 – schematic representation of folic acid grafted CdSeTe/ZnS SNCs. B2 – observation of SNCs accumulation dynamics in the brain region over a course of 42 days after intrathecal administration. Adapted with permission from ref. 32. Copyright 2019 American Chemical Society.

Deep-tissue imaging with PbS/CdS and PbS/CdS/ZnS SNCs and its subsequent potential to aid in diagnosis have been demonstrated on numerous occasions.^26–29^ In a recent example, Tian *et al.* imaged sentinel tumor lymph nodes (LN) with PbS/CdS SNCs (Fig. 3 – A).^30^ Sentinel LNs play a key part in tumor metastasis, so their excision and biopsy is common in tumor staging, treatment planning, and prognosis. Detection and excision of sentinel LNs is aided *via* preoperative lymphoscintigraphy with technetium-99m, or alternatively, with clinically approved PL molecule indocyanine green (ICG).^31^ Although the latter avoids the need for radioactive agents, it still suffers from photobleaching, shallow imaging depth, and poor imaging contrast. Thus, there is an incentive for better suited lymphoscintigraphy PL contrast agents capable to detect metastasis in sentinel LNs during their intraoperative excision. In their study, the authors imaged primary metastatic tumor and sentinel LNs simultaneously with the help of a custom-synthesized donor–acceptor–donor (D–A–D) infrared fluorescent dye (IR-FD) and PbS/CdS SNCs, respectively (Fig. 3 – A1). The PL spectra of the contrast agents were well spectrally separated, peaking around 1100 nm for the IR-FD and 1600 nm for SNCs, which allowed for simultaneous NIR-II PL imaging of different targets. To improve biocompatibility, PbS/CdS SNCs were modified with a dense polymer coating and grafted with anti-CD3 antibody for increased selectivity against sentinel LNs. Benchmarked against ICG in *ex vivo* experiments, PbS/CdS SNCs showed prominently greater PL imaging depth and signal-to-noise contrast. In fact, to attain similar imaging contrast when detecting popliteal and sacral LNs in nude mice, the ICG concentration needed to be several orders of magnitude greater than that of PbS/CdS SNCs. Even at 40 pmol administered concentration, PbS/CdS SNCs provided high-contrast imaging for PL-guided removal of the popliteal LNs. Furthermore, the NIR-II PL of SNCs allowed resolving structures below 100 μm, which aided in the recognition of LNs against the muscle background. On the other hand, the unique structure of the PEGylated (grafted with polyethylene glycol - PEG) IR-FD dye endowed it with high PLQY (∼6%) and long circulation time in the bloodstream, allowing it to be accumulated and observed in U87MG tumor-bearing mice. The dual imaging of tumors and LNs was tested in mice bearing U87MG and 4T1 cancer models. The IR-FD dye would be administered first to coarsely visualize all tumor sites, primary and metastatic, while subsequently injected PbS/CdS SNCs clearly demarcated the sentinel LNs (Fig. 3 – A2). Tumor-invaded LNs could be then excised, maximally eliminating risks of metastasis and relapse after the primary tumor removal (Fig. 3 – A2). Owing to the all-NIR operation of the IR-FD dye and PbS/CdS SCNs, tumor and LNs can be visualized intraoperatively under room lights, making it possible to carry out surgical tasks under normal lighting conditions. Besides the clearly improved intraoperative lymphoscintigraphy and image-guided surgery, authors note that NIR-II contrast agents, like PbS/CdS SNCs, could help to better understand the biological events that underline the metastasis of tumors.

Although Cd-based SNCs have their emission mostly confined to the visible spectral range (bulk CdSe band gap being 1.7 eV), Cd-based SNCs emitting in NIR-I can be used for subcutaneous imaging. Liang *et al.* prepared CdSeTe/ZnS SNCs, modified with PEGylated amphiphilic polymer and folic acid (FA), as glioma imaging agents (emission peak around 800 nm) (Fig. 3 – B1).^32^ Nude mouse models bearing U87MG glioblastoma were intrathecally injected with PEG- and FA-grafted SNCs or PEG-only ones as a control. After injection, the PEG-only CdSeTe/ZnS SNCs distributed across the mouse body and showed no preferential accumulation in the tumor even after 14 day period. On the contrary, PEG- and FA-grafted SNCs showed selective accumulation in the brain tumor after 2 days, and their PL signal could be observed throughout the 42 day period (Fig. 3 – B2). Prolonged imaging allowed to observe PL signal intensity fluctuation as in flow and ebb tides, with a period of 6–8 days, that is believed to be related to the flow and ebb of the cerebrospinal fluid movement.^33^ Whole brain PL imaging with micrometer precision was also showcased by Shi *et al.*, who used improved and photostable 1630 nm emitting Zn^2+^-doped PbS SNCs to image cerebral vasculature networks through intact scalp.^34^

Small size and bright PL in NIR-I and NIR-II of Cd- and Pb-based SNCs make them potent candidates to study NP/drug pharmacokinetics in cerebrospinal fluid of the brain, brain lesion formation, and detection of its early onsite markers, as well as observation of blood perfusion in cerebral vessels *via* non-invasive PL imaging in the NIR.

## 3. Cu- and In-based SNCs

As discussed in Section 2, Pb- and Cd-based SNCs are the staple as far as the brightness is concerned. However, the presence of intrinsically toxic elements (*i.e.*, cadmium and lead) in their composition represents a roadblock towards their application. While this aspect is of limited concern for *in vitro* and preclinical applications, it is unlikely that heavy-metal-containing SNCs will ever be injected in humans.^35^ Moreover, processes involved in the preparation and disposal of such SNCs might have an impact on the environment and the wildlife. To allay these concerns, heavy-metal-free SNCs are sought-after materials as possible contrast agents for different optical imaging modalities.

In particular, copper- and indium-based SNCs have been the subject of intense investigation. SNCs made of CuIn(S,Se)_2_, Cu_2−*x*_(S,Se)_*x*_, InP, and InAs being arguably among the most relevant examples in this context. While In^3+^ is considered a non-toxic metal ion, Cu^+/2+^ is to some extent cytotoxic.^36^ Therefore, it is debatable whether copper-based SNCs could be considered intrinsically biocompatible.^37^

Given the different optical properties featured by the above-mentioned SNCs, it is useful to split the discussion into two separate blocks: one dealing with plasmonic SNCs and the other discussing luminescent SNCs.

### 3.1 Plasmonic Cu-based SNCs

Copper chalcogenide (Cu_2−*x*_E, where E = S, Se, Te and *x* = 1–2) SNCs have emerged as an alternative to noble-metal-based plasmonic materials. In Cu_2−*x*_E SNCs, the localized plasmon resonance (LSPR) arises from the collective motion of vacancies rather than electrons.^38^ These vacancies are localized at the top of the valence band, and they originate from the defective stoichiometry displayed by some of the compositions. For example, Cu_2_S (chalcocite crystal structure) is a fully stoichiometric material and does not generally display LSPR, but rather NIR-II PL.^39^ On the contrary, CuS (covellite crystal structure) showcases strong NIR-centered LSPR. Although the absorption cross section of these materials is smaller than the one displayed by metallic plasmonic NPs based on, *e.g.*, gold or silver (10^−14^ to 10^−13^ cm^2^*vs.* 10^−11^ to 10^−9^ cm^2^), they have the unique advantage of featuring LSPR peaks across NIR-I and NIR-II even at sizes as small as few nanometers. This characteristic makes them attractive contrast agents for NIR-based imaging techniques, as well as light-to-heat converters for photothermal therapy (PTT).^40^

**Fig. 4:**
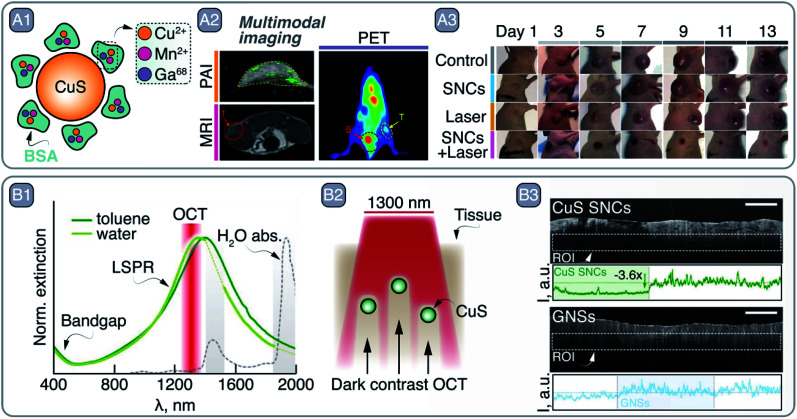
A – multimodal imaging and PTT with CuS SNCs: A1 – schematic representation of CuS SNCs coated with Cu^2+^-, Mn^2+^-, and Ga68-loaded BSA. A2 – multimodal imaging capabilities of these CuS SNCs demonstrated by imaging SKOV-3 tumor in Balb/c mouse *via* PA imaging (PAI), MRI, and PET. A3 – PTT capabilities of CuS SNCs under 980 nm excitation. Adapted from ref. 41, Copyright 2018, with permission from Elsevier. B – CuS SNCs as dark contrast agents in OCT: B1 – absorption profile of covallite CuS SNCs in water and toluene. Major water absorption bands as well as OCT imaging wavelength are indicated. B2 – representation of pork fat tissue experiment highlighting CuS as dark contrast agents for OCT. B3 – experimental imaging data of B2, comparing dark and light OCT contrast agents – CuS and gold nanorods (GNRs), respectively. Adapted from ref. 43 with permission from John Wiley and Sons, copyright 2020 (CC BY 3.0).

To that end, Zhou *et al.* developed 5 nm Mn-doped CuS SNCs with broad LSPR centered at 1000 nm.^41^ The small size of the SNCs and their bovine serum albumin (BSA) coating (Fig. 4 – A1) made them biocompatible and clearable *via* renal excretion. Moreover, upon modification of the SNCs with ^68^Ga – a short-lived radioisotope – the NPs could act as contrast agents for three different imaging modalities, namely MRI, PA imaging, and PET (Fig. 4 – A2). The photothermal conversion capabilities of the SNCs were exploited to perform *in vivo* PTT on a SKOV-3 tumor-bearing Balb/c mouse (Fig. 4 – A3). Similarly, Cu_3_P SNCs were developed as multimodal imaging and theranostic agents, combining MRI and PA imaging with PTT and photothermally-enhanced chemodynamic therapy.^42^

Along these lines, in a study published last year, some of us demonstrated the potential of CuS SNCs as negative contrast agents in OCT.^43^ Upon exploring the optical properties of a library of Cu_2−*x*_S SNCs, 25 nm sized nanoplates were selected as the best candidates for this application. The lack of scattering from these small CuS SNCs at the OCT imaging wavelength (*i.e.*, 1300 nm, well within the LSPR, see Fig. 4 – B1) ensures that all extinguished probing photons are absorbed. As a result, CuS SNCs could be localized *ex vivo* within highly scattering tissues, such as pork fat, thanks to the darkening of the OCT images (Fig. 4 – B2–3), a result that opened the door to the future application of this imaging modality for a more effective OCT-based diagnosis of atheromatous plaques.

### 3.2 Photoluminescent Cu- and In-based SNCs

As mentioned earlier, Cu_2_S SNCs do not display LSPR, but rather PL in the NIR-I. Li *et al.* recently employed ultrasmall, Zn^2+^-doped, glutathione-coated Cu_2_S SNCs as NIR PL contrast agents.^44^ Under 365 nm excitation (admittedly not ideal to address NIR contrast agents subcutaneously), the SNCs featured emission centered at 700 nm and were preferentially taken up by cancer cells, wherein H_2_O_2_ is present at higher concentration than in healthy cells. The authors went on to prove that Cu_2_S:Zn^2+^ SNCs produce OH radicals in the presence of H_2_O_2_, justifying the cytotoxic effect preferentially induced in tumor cells. This chemodynamic therapeutic effect was later employed *in vivo* to suppress the tumor growth in Balb/c mice with a 56% inhibition rate compared to a control group treated with saline.

**Fig. 5:**
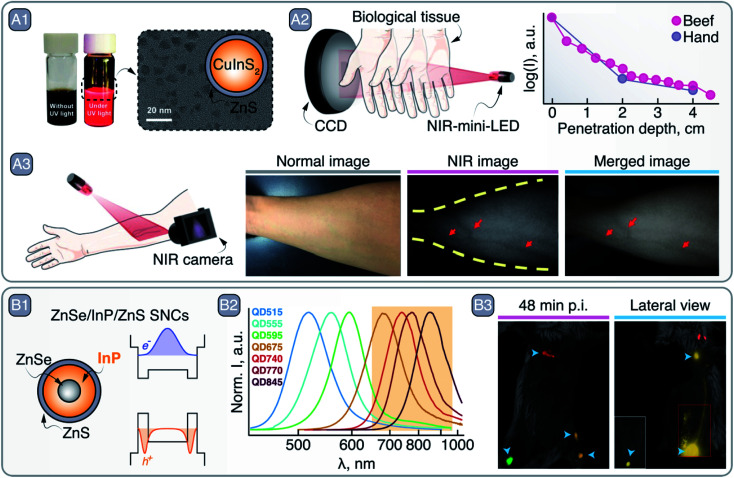
A – CuInS_2_/ZnS SNCs as NIR-emitting mini-LEDs: A1 – bright field and NIR PL photographs of CuInS_2_/ZnS SNC dispersions, as well as their TEM image and graphical representation. A2 – NIR PL of CuInS_2_/ZnS SNCs penetration through tissues. A3 – vein imaging with CuInS_2_/ZnS SNCs mini-LED. Adapted with permission of Royal Society of Chemistry, from ref. 52; permission conveyed through Copyright Clearance Center, Inc. B – ZnSe/InP/ZnS SNCs as NIR imaging agents: B1 – schematic of the core/shell/shell ZnSe/InP/ZnS SNCs and their bandgap alignments with corresponding electron and hole wave functions. B2 – change in the PL spectrum peak position of ZnSe/InP/ZnS SNCs as a function of InP deposition. B3 – left inguinal LN, axillary LN, and right inguinal LN and popliteal LN multiplexed imaging with different ZnSe/InP/ZnS SNCs 48 min post injection (p.i). Lateral view, 1 h p.i., shows SNCs lymphatic drainage from the inguinal LN to the axillary LN; inset image with higher thresholding shows the contrast localized in the inguinal LN without image saturation. Adapted with permission from ref. 55. Copyright 2021 American Chemical Society.

Another class of copper-based luminescent SNCs is that of I–III–IV CuIn(S,Se)_2_. Their synthesis has been developed relatively recently, hence there is ample room for improvement in terms of control of their morphology, core/shell architecture,^45^ and size dispersion. The latter is an issue when aiming for biological applications due to the different fate experienced in the body by NPs of different sizes.^46^ Controlling the synthesis of ternary CuIn(S,Se)_2_ SNCs requires great care, since the reactivity of Cu^+^ and In^3+^ is different, and thus fine-tuning of the reaction mixture composition is necessary. The emission of CuIn(S,Se)_2_ SNCs is within the visible or NIR spectral range (750–1300 nm),^47–50^ and arises from carrier recombination events that involve copper-related interbandgap states and strong electron-phonon coupling.^51^ The non-excitonic nature of the emission translates to a large Stokes-shift and PL lifetimes up to several hundreds of nanoseconds – 1–2 orders of magnitude longer than the excitonic emission typical of PbS and CdSe SNCs. This extended lifetime was harnessed by Pons *et al.* to perform *in vivo* imaging of single cells in the bloodstream. The authors prepared alloyed Cu–Zn–In–Se SNCs shelled with ZnS and modified their surface with poly(imidazole-*b*-sulfobetaine) block polymer.^47^ The SNCs boasted an 800 nm emission under 660 nm excitation, PLQY as high as 30% in water, and lifetime on the order of 150–300 ns. After labelling rat red blood cells (RBCs) with SNCs *via* pinocytosis and electroporation, the cells were re-injected into the animal. The authors microscopically imaged single RBCs in small vessels (few micrometers in diameter) with slow blood flow. A20 lymphoma cells – a type of circulating tumor cells (CTCs) – were also labelled and imaged in larger blood vessels after injection into the animal, enabling the estimation of their concentration in the bloodstream as well as the local blood flow. A similar 800 nm emission was featured by the CuInS_2_/ZnS SNCs prepared by Huang *et al.*, who pushed the emission of these SNCs towards the NIR by using InI_3_ as the indium source instead of the more commonly employed In-acetate.^52^ Upon growing the ZnS shell, partial etching of the CuInS_2_ core was observed (owing to the typical Cu^+^/In^3+^-to-Zn^2+^ exchange in this system), resulting in an overall blue-shift of the emission in hexane (from 933 to 813 nm) and PLQY of 94.8% (Fig. 5 – A1). These SNCs were coated on a blue-emitting LED chip to prepare a NIR-emitting mini-LED, which featured an emission capable of penetrating 4.5 cm of tissue (Fig. 5 – A2). Upon illuminating a human arm with the mini-LED, and using a NIR camera to detect the reflected light, the veins could be precisely located thanks to their darker appearance that follows from deoxyhemoglobin absorption in NIR-I (Fig. 5 – A3). Very recently, core/shell CuInSe_2_/ZnS SNCs with NIR-II emission were reported by Lian *et al.*^53^ The authors controlled the position of the emission peak of these SNCs by tuning the nominal Se/In ratio between 0.5 and 4. After the growth of a ZnS shell and transfer to water with the aid of PEGylated phospholipids, the optimized SNCs featured an emission centered at 1050 nm and a PLQY of 21.8%. Modification of their surface with anti-EpCAM antibody afforded SNCs selective targeting towards human breast cancer cells. *In vivo* imaging was also performed in a mouse, observing preferential accumulation of antibody-modified SNCs at the tumor site, with a maximum signal at the tumor appearing 8 h post injection. In another study, CuInSe_2_/ZnS:Al SNCs were incorporated into microneedle arrays.^54^ By virtue of NIR imaging, information from within the skin could be retrieved from the microneedle patches over the period of 9 months, opening up new avenues for “on-body” information storage and biosensing.

Despite these results, an important limitation of CuIn(S,Se)_2_-based SNCs is the difficulty to push their emission beyond 1300 nm.^48^ On the contrary, this could be accomplished with In-based binary SNCs containing pnicogens (P, As, Sb), whose emission can span the whole 500–1600 nm range.^55–58^ The chemistry of these SNCs is yet less explored than the one of CuIn(S,Se)_2_ SNCs and their synthesis usually involves long reaction times and toxic, oxygen-sensitive precursors such as pyrophoric tris(trimethylsilyl/germanyl) arsine or metal hydrides.^56^ The Bawendi group proposed an alternative and safer, redox-based process where In^+^ precursors are used along with As-amines. Unfortunately, the reported PLQY of so-produced InAs SNCs is below 1%, overcoming 12% only after the growth of a CdSe shell. Clearly, there is plenty of room for improvement in the synthesis processes of In-based SNCs.

The emission mechanism in III–V In–SNCs remains a matter of investigation. Electron-phonon coupling seems to play a relevant role in determining the broadband emission spectrum, along with carrier recombination events involving trap states,^59^ similarly to what has been reported for CuIn(S,Se)_2_ SNCs. Strategies to increase the brightness of these SNCs include the growth of several shells of controlled composition,^57,60,61^ and treatment with HF to passivate In^3+^-related surface trap states.^62,63^ Doping with Cu^+^ was also proposed to tune the emission of InP SNCs towards the NIR, harnessing the energy transfer from the semiconductor matrix to the transition metal.^64,65^

Although low PLQY values are generally reported for these SNCs, their optical versatility and inherent non-toxic nature make III–V In–SNCs amenable to applications in medicine. For example, in a recent study, Saeboe *et al.* performed multiplexed *in vivo* imaging in NIR-I using ZnSe/InP/ZnS SNCs (Fig. 5 – B).^55^ The clever core/multi-shell architecture and the resulting band scheme induced localization of the electron's wavefunction at the ZnSe core and the holes at the first InP shell, in turn pushing the emission of InP deeper into the NIR (Fig. 5 – B1). By tuning the thickness of the InP shell, the emission maximum was varied from 369 to 845 nm (Fig. 5 – B2). After modification of the surface with phospholipids, SNCs of different sizes with emission between 650 and 1000 nm were injected in different sites of a BALB/c mouse to visualize lymphatic drainage at the whole-body level *via* multiplexed PL imaging (Fig. 5 – B3).

As can be seen, venture of Cu- and In-based SNCs into the NIR PL imaging is still in its infancy, however the high extinction coefficient associated with these structures makes them readily available for dark contrast OCT and PA imaging, as well as PTT. Furthermore, these materials are promising substitutes to organic dyes and Pb- and Cd-based SNCs in research-oriented *in vitro* and *in vivo* studies.

## 4. Ag-based SNCs

Silver chalcogenide (Ag_2_S, Ag_2_Se, and Ag_2_Te) SNCs have recently came to light in bio-oriented nanotechnology research as a direct response to Cd- and Pb-based SNCs, meeting many of the pre-requisites of deep-tissue PL imaging in the NIR.^66^ Despite being Cd^2+^ and Pb^2+^ free, Ag^+^ can be cytotoxic, yet low solubility of Ag_2_S ensures negligible release of free Ag^+^ into the biological milieu and reduces concerns related to the silver poisoning (*i.e.*, argyria). In its bulk form, Ag_2_S has a direct band gap of 1 eV, thus having large extinction coefficient for wavelengths greater than 850 nm, relevant for NIR excitation.^67^ Furthermore, Ag_2_S emission usually peaks around 1200 nm, and due to the small Bohr exciton radius (∼2 nm) of the material, Ag_2_S SNCs larger than 4.5 nm have their PL band centered in the NIR-II. Despite certain difficulties associated with the synthesis of high-purity and high-PLQY Ag_2_S SNCs,^68,69^ the PL band is practically insensitive to the dimensions of these SNCs and guarantees NIR-II emission for nanocrystals >4 nm in diameter. However, the synthetic route employed in the preparation of Ag_2_S SNCs seems to have a large effect on their spectral tunability.^68^ Similarly to Ag_2_S, Ag_2_Se SNCs can be made to exhibit PL in the 1000–1600 nm range, and are also potent NIR imaging contrast agents.

**Fig. 6:**
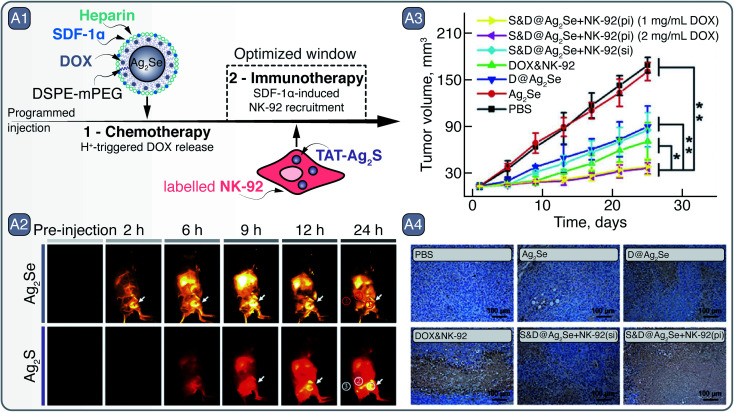
A – Tumor recognition and eradication by a collaboration of S&D@Ag_2_Se and Ag_2_S-labeled NK-92 cells: A1 – depiction of S&D@Ag_2_Se SNCs and Ag_2_S-labelled NK-92 cells as well as the principle of programmed injection that takes advantage of chemotherapy and immunotherapy facilitated by the respective agents. A2 – time dependent imaging of MDA-MB-231 tumor bearing nude mouse after programmed injection of S&D@Ag_2_Se SNCs and Ag_2_S-labeled NK-92 cells. A3 – reduction in tumor volume after treatment with of S&D@Ag_2_Se SNCs and Ag_2_S-labeled NK-92 cells, as well as their analogs as controls. Distinctly, programmed injection (pi) yielded greater tumor reduction than simultaneous injection (si) of S&D@Ag_2_Se SNCs and Ag_2_S-labeled NK-92 cells. A4 – terminal deoxynucleotidyl transferase-mediated dUTP nick-end labeling of the deep region of tumors after treatment. Adapted from ref. 70 with permission from John Wiley and Sons, copyright 2018 (CC BY 3.0).

In the study by Hao *et al.*, both Ag_2_S and Ag_2_Se SNCs were combined to showcase the multiplexed NIR imaging of these SNCs and how they can aid in developing a programmed, combined chemotherapy and immunotherapy (Fig. 6 – A).^70^ Ag_2_Se SNCs (S&D@Ag_2_Se) with emission around 1350 nm were coated with PEGylated phospholipids, heparin, and stromal-cell-derived factor-1α (SDF-1α), and loaded with chemotherapeutic drug doxorubicin (DOX). Meanwhile, TAT – cell-penetrating peptide – grafted Ag_2_S SNCs (TAT-Ag_2_S; PL ∼1050 nm) were preloaded into natural killer cells (NK-92) destined for adaptive cellular immunotherapy. The premise of this therapeutic approach relied on the initial accumulation of S&D@Ag_2_Se in the tumor, where under low pH the DOX could be released; at the same time, SDF-1α would act as a beacon^71^ – selectively guiding the NK-92 cells towards the tumor site to induce cancer cell death (Fig. 6 – A1). The multiplexed imaging with Ag_2_S and Ag_2_Se in the NIR-II helped to assess the guidance of NK-92 cells towards the tumor *in vivo*. S&D@Ag_2_Se were first injected into the MDA-MB-231 tumor-bearing nude mice *via* tail vein, and their accumulation at the tumor site was observed over a 24 h period. The pharmacokinetic study with S&D@Ag_2_Se allowed to identify the time window when to administer the NK-92 cells; specifically, TAT-Ag_2_S labeled NK-92 cells were injected into mice 6 h after the S&D@Ag_2_Se administration. With the help of TAT-Ag_2_S labeling, accumulation of NK-92 cells at the tumor site could be observed subcutaneously *via* NIR imaging. Guided by the tropism effect of SDF-1α, pre-delivered by the S&D@Ag_2_Se SNCs, NK-92 cells at the tumor were first observed 3 h post injection and showed further gradual accumulation at the site of interest (Fig. 6 – A2). In contrast, simultaneously administered S&D@Ag_2_Se and TAT-Ag_2_S labeled NK-92 cells showed the NIR emission in tumors only from the S&D@Ag_2_Se, while the NK-92 cells were likely eliminated from the bloodstream due to the relatively low half-life. Finally, the programed, combined chemotherapy and immunotherapy was shown to effectively suppress tumor growth in orthotopic mouse model with xenografted MDA-MB-231-Luc cells even at a low DOX loading doses of 1 mg mL^−1^, all due to optimized administration of therapeutics aided by NIR imaging with Ag-based SNCs (Fig. 6 – A3–4).

Tumor targeting and eradication with Ag_2_S SNC has also been recently demonstrated by Wang *et al.*, who used Ag_2_S SNCs exhibiting NIR-I PL and significant light-to-heat conversion efficiency employed for imaging and PTT, respectively.^72^ The surface of Ag_2_S SNCs was modified with AMD3100, a CXCR4 receptor antagonist, aimed at identification and tracking of breast cancer metastasis *in vivo*. The AMD3100 modified Ag_2_S SNCs helped to visualize and distinguish between low and high CXCR4 expressing tumors *in vivo*, MCF-7 and 4T1, respectively, and to predict the risk of metastasis by means of NIR imaging. Mimicking late-stage breast cancer and its metastasis, orthotopic transplantation of 4T1 cells was established in nude mice and the invasion of primary tumor from the mammary fat pads into the lungs was observed over a period of 20 days. The photothermal effect of Ag_2_S SNCs was exploited to reduce the spread of metastasis and eradicate the primary tumor.

In a similar vein, Ling *et al.* devised an intricate theranostic system self-assembled out of Fmoc-His peptide, mercaptopropionic acid-functionalized Ag_2_S SNCs (MPA-Ag_2_S), DOX, and a small molecular NIR absorber A1094.^73^ Er^3+^ bait-ions were introduced to cross-link histidine imidazole groups in Fmoc-His and carboxyl groups in the MPA-Ag_2_S SNCs, while DOX and A1094 facilitated the multi-component self-assembly by a number of intramolecular interactions. As prepared, this theranostic system is non-photoluminescent, due to Ag_2_S PL quenching by A1094, but at low pH conditions the system disassembles prompting rapid PL response from Ag_2_S SNCs and DOX release. The importance of site-specific PL turn ON systems lies in their ability to only image the area of interest, increasing the signal-to-background ratio and accurately delineating the diseased tissue. In the work by Li *et al.* response to ONOO^−^ was used to bleach the A1094 conjugated on the surface of Ag_2_S SNCs together with VCAM1 binding peptide.^74^ In the presence of ONOO^−^, found at sites of traumatic brain injury, the PL quenching of Ag_2_S by A1094 would cease, allowing high contrast NIR-II PL imaging of the injured area.

Furthermore, Ag-based SNCs serve as NIR PL contrast agents in multimodal imaging systems. For instance, Ag_2_Se SNCs coupled with gadopentetic acid (Gd-DTPA), a clinically approved MRI contrast agent, could be used for PL as well as MR imaging,^75^ whereas Cu^+^-doped Ag_2_S SNCs can achieve NIR PL and PA imaging, as well as PTT.^76^ The presence of Cu^+^ in Ag_2−*x*_Cu_*x*_S SNCs induced a bathochromic shift of the emission peak from 765 to around 820 nm and granted the SNCs a light-to-heat conversion efficiency up to 44%, successfully utilized by both PA imaging and PTT.

**Fig. 7:**
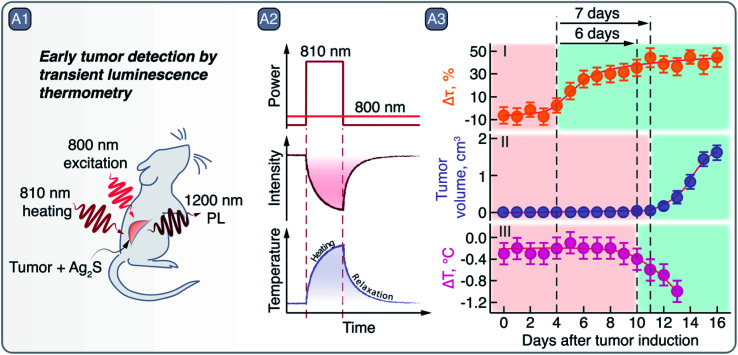
A – Early tumor detection by Ag_2_S SNCs as luminescent thermometers: A1 – schematic representation of the experiment in which Ag_2_S SNCs were used as NIR PL thermometers under 800 nm excitation after a mild heating (using 810 nm laser radiation) of the area of interest. A2 – qualitative depiction of Ag_2_S PL quenching after 810 nm laser excitation and the tissue temperature increase and relaxation, measured non-invasively *via* 1200 nm emission of Ag_2_S. A3 – comparison of early tumor detection *via* transient PL thermometry (I), visual inspection (II), or thermographic imaging (III). Transient PL thermometry provides evidence of tumor 6 and 7 days earlier than visual or thermographic inspections, respectively. Adapted from ref. 77 with permission from John Wiley and Sons, copyright 2018 (CC BY 3.0).

Regarding light–heat interaction, thermally-induced changes in the PL band profile or intensity of Ag_2_S SNCs can be used to measure local temperature non-invasively and conduct multivariate diagnostics. Santos *et al.* showed that NIR-II PL of Ag_2_S SNCs can be used not only for tumor visualization, but also to more accurately discriminate between healthy and malignant tissues by means of PL thermometry (Fig. 7 – A1).^77^ After correlating the PL intensity change of Ag_2_S SNCs with temperature, the transient thermal relaxation profiles of tissues could be acquired after mild laser heating (Fig. 7 – A2). By measuring these thermal relaxation profiles of healthy and cancerous tissue in a murine model of melanoma, the authors demonstrated the capacity of PL thermometry to detect the onset of tumor development 6 days before it could be observed visually or through thermographic imaging (Fig. 7 – A3). Our group has also exploited PL thermometry properties of Ag_2_S SNCs to detect minute temperature changes in the brain during a barbiturate-induced coma. These changes can be associated with brain activity, monitored non-invasively through skull and scalp in real-time thanks to the NIR-II PL imaging.^78^

Despite great progress in the use of Ag-based SNCs in biomedical research,^73^ their low PLQY often limits their applicability. Novel nanocrystal preparation techniques have been proposed to address this problem. Santos *et al.* used ultrafast laser irradiation to form a protective AgCl layer over Ag/Ag_2_S SNCs, increasing their PLQY up to 10.7%.^79^ Alternatively, Ag_2_Te/Ag_2_S core/shell SNCs with 4.3% PLQY and emission tunable in 1300–1560 nm range were made by the Wang group.^80^ Incorporation of Pb^2+^ into Ag_2_Se has also shown to improve the brightness of these SNCs.^81^ However, without falling back onto toxic heavy metals, H. Yang, have made alloyed AgAuSe SNCs with emission tunable in the 820–1170 nm range and PLQY up to 65.3%.^82^ And most recently, Au-doped Ag_2_Te SNCs have been applied to study and diagnose ischemia.^83^ Thus, the progress made on the use of Ag-based SNCs in multivariate diagnostics and theranostics, complemented with the improvement in the brightness of these nanocrystals, distinguishes these materials as potent candidates for further biomedical exploration.

## 5. RENPs

RENPs bring together highly desired optical properties for NIR bioimaging and multivariate diagnostics. First, they feature very long-lived emissions, in the range of μs to ms (in contrast to the ns- or μs-long PL associated with the best performing SNCs), which easily allow for time-gated imaging and lifetime barcoding.^84^ Second, they often show large spectral separation between excitation and emission wavelengths, resulting in clearly discernible emission bands with reduced interference from both the excitation source and tissue autofluorescence. Third, RENPs allow diverse emission tunability within the NIR-I and NIR-II, by simply selecting appropriate lanthanide ions (Ln^3+^) as dopants (*e.g.*, Tm^3+^, Nd^3+^ and Er^3+^ feature emissions overlapping with the 800–1600 nm range). Fourth, their emissions present narrow bandwidths, reducing the possibility of channel crosstalk during spectral multiplexing. Finally, their luminescent properties stand out among other PL materials due to their high resistance to photoblinking and photobleaching. Besides emission in the NIR, rationally designed RENPs allow for upconversion PL – emission of photons of higher energy than initially absorbed ones. Nevertheless, this comes at a cost in the form of low radiative transition probabilities, yielding poor Ln^3+^ molar extinction coefficients on the order of 0.1–10 M^−1^ cm^−1^.^85^ Although different strategies aimed to improve the absorption (that translates to brightness) of RENPs have been developed, it still represents an important bottleneck for these nanomaterials.^85,86^

Generally, RENPs are the result of embedding Ln^3+^ within the crystalline structure of a proper dielectric host material which has three main fundamental roles: (i) crystal structure asymmetry and crystal field can be used to increase the probability of the 4f–4f transitions; (ii) low cutoff phonon energies reduce non-radiative quenching of the excited states, and (iii) acting as a platform where different sensitizers and activators can be combined to achieve specific luminescent features.^87–89^

RENPs can be produced by multiple approaches, which can be generally split into syntheses performed in organic solvents or aqueous media. Thermal decomposition is a common method to produce RENPs due to its relative simplicity, consisting of the decomposition of organo-metallic Ln^3+^ precursors at high temperatures. This synthetic strategy is closely followed by the thermal co-precipitation, which is associated with relatively non-toxic precursors and milder conditions, although it involves a larger number of synthetic steps. These methods offer high synthetic control, allowing tunability of size, shape and nano-architecture, size monodispersity, and high crystallinity of the resulting RENPs.^90,91^

**Fig. 8:**
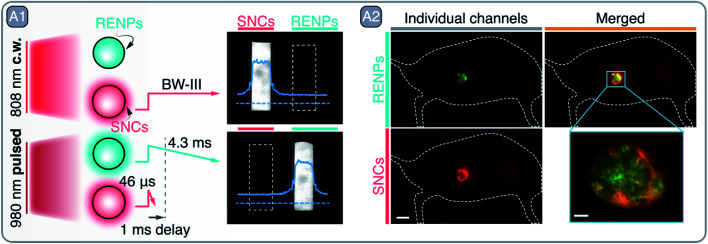
A – Multiplexed imaging with RENPs and SNCs: A1 – graphical depiction of RENPs and SNCs PL behavior under continuous wave (c.w.) 808 nm and pulsed 980 nm excitation. NIR images of RENPs and SNCs dispersions under c.w. and pulsed excitation are also shown, sole detection of RENPs PL under pulsed 980 nm excitation is achieved by implementing 1 ms delay, which filters out short-lived PL of SNCs. A2 – two-plexed imaging of CT-26 tumor with RENPs and SNCs. Adapted by permission from Springer Nature: Springer Nature Nature Biotechnology ref. 96, © 2019.

The stunning potential of RENPs operating in the NIR-I and NIR-II is not only reflected in the abundance of works related to *in vivo* bioimaging, but in the number of those that explore their PL properties for novel applications in the fields of drug delivery, multivariate diagnostics, and theranostics.^92–95^ Zhong *et al.* combined RENPs and PbS SNCs in a multiplexed diagnostic strategy, that also offered therapeutic properties, due to their functionalization with immunomodulating antibodies (Fig. 8 – A).^96^ In this work, the authors optimized the RENPs to achieve maximum emission within the NIR-II (1550 nm), where autofluorescence is negligible and the light penetration depth into tissues is maximized. Alpha-phase NaYbF_4_: 2 mol% Er^3+^, 2 mol% Ce^3+^, 10 mol% Zn^2+^/NaYF_4_ RENPs showcased an absolute PLQY of ∼5% and an ∼11-fold increase in their emission at 1550 nm over previous beta-phase compositions.^97^ The subsequent surface functionalization of the RENPs with hydrophilic and cross-linked coating layers provided water dispersibility and biocompatibility, and resulted in a biliary excretion of up to 90% of the injected RENPs in a two week period. Further surface modification allowed to attach anti-PD-L1 mAb (atezolizumab) to the RENPs for the specific targeting of CT-26 colon cancer cells, which over-express PD-L1 ligand, responsible for the evasion of the immune system. The remarkable long PL lifetime exhibited by these RENPs (4.3 ms) allowed for easy *in vivo* multiplexed imaging when combined with PbS SNCs (emission ∼1600 nm). PbS SNCs exhibited a lifetime of 46 μs upon excitation at 980 nm (Fig. 8 – A1) and were able to specifically label CD8^+^ T cells *in vivo* after bioconjugation with anti-CD8α mAb. Notably, CD8^+^ T cells could be tracked by anti-CD8α-conjugated PbS SNCs without interference from the RENPs emission by using excitation at 808 nm (Fig. 8 – A2). Meanwhile, the successful labeling of the CT-26 tumors by the RENPs could be visualized excluding the PbS SNCs emission when 1 ms excitation pulses at 980 nm were used and emission detection was time-gated. The migration of CD8^+^ T cells towards the tumor was correlated with the blockade of PD-L1 receptors from CT-26 tumor cells, caused by the RENPs-*anti*-PD-L1. As a consequence, the immune evasion by the tumor cells was hampered, and the CD8^+^ T cells were able to recognize the tumor and to migrate from the periphery towards its center. Remarkably, this process could be monitored *in vivo* by using the proposed two-plexed NIR imaging with PbS SNCs and RENPs (Fig. 8 – A2).

**Fig. 9:**
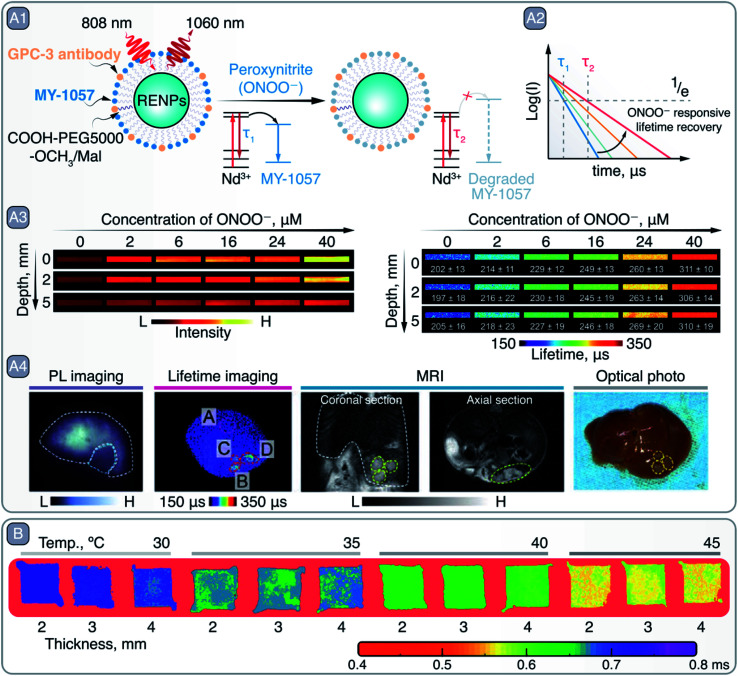
A – Lifetime-based detection of peroxynitire (ONOO^−^) and hepatocellular carcinoma imaging with RENPs: A1 – schematic representation of RENPs engineered for ONOO^−^ sensing. A2 – qualitative depiction of Nd^3+ 4^F_3/2_ excited state lifetime restoration (measured *via* 1060 nm emission) due to MY-1057 degradation in the presence of ONOO^−^. A3 – intensity- (left) and lifetime-based (right) imaging of capillaries containing RENPs as a function of ONOO^−^ concentration and imaging depth. A4 – PL intensity- and lifetime-based imaging, as well as MRI, of a mouse bearing multiple hepatocellular carcinoma lesions, and optical photo of the dissected liver. Adapted from ref. 98 with permission from John Wiley and Sons, copyright 2020 (CC BY 3.0). B – lifetime-based PL thermometry with RENPs at various imaging depths and temperatures. Adapted from ref. 102 with permission from John Wiley and Sons, copyright 2020 (CC BY 3.0).

One major limitation of NIR bioimaging, when used as a tool for quantification, is the unavoidable attenuation of PL when propagating through tissues. This can be difficult to predict as it depends on factors such as tissue composition and thickness. Nevertheless, these pitfalls can be partially overcome by relying on PL lifetime-based bioimaging. Zhao *et al.* used this approach for *in vivo* bioimaging of hepatocellular carcinoma, while simultaneously achieving detection and reliable quantification of ONOO^−^ within the tumoral microenvironment (Fig. 9 – A).^98^ Researchers created a Föster resonance energy transfer (FRET) system working in NIR-II that combined Nd^3+^-doped core/shell RENPs as FRET donors and a cyanine dye (MY-1057) grafted to their surface as an energy acceptor (Fig. 9 – A1). After 808 nm excitation, Nd^3+^ within the RENPs relax non-radiatively to their ground state by transferring the energy from the ^4^F_3/2_ excited state to the MY-1057, which is reflected in the reduced lifetime of the ^4^F_3/2_ → ^4^I_11/2_ (∼1060 nm) radiative transition. Yet, the ONOO^−^ dependent degradation of MY-1057 resulted in diminished FRET and consequently in an increase in the lifetime of the 1060 nm emission (Fig. 9 – A2), used for ONOO^−^ detection. This lifetime-based quantification strategy was superior to the traditional steady-state PL approach (Fig. 9 – A3), demonstrating the enhanced reliability of the lifetime-based technique at imaging depths up to 5 mm. Furthermore, the detection of hepatocellular carcinoma lesions within the liver of mice was demonstrated using this strategy. ONOO^−^ induced a change in the lifetime of the RENPs specifically within the tumor regions, which permitted to visualize tumor areas that could not be otherwise detected using traditional steady-state PL imaging (Fig. 9 – A4).

Similar *in vivo* multivariate diagnosis has been pursued by other authors, but relying on analyte quantification through steady-state ratiometric PL strategies: one emission band of the RENPs may remain constant and is used as a reference, while another one varies in intensity due to the presence of the target analyte.^93,98–101^ It is important to highlight that these works focus on detecting analytes that can cause cellular oxidative stress (*e.g.* metformin) or are result-products of it, such as peroxynitrite, hypochlorous acid, or hydrogen peroxide.^93,98–101^ As a consequence, their oxidative properties are used to chemically modify the FRET acceptor, usually a cyanine dye that acts by quenching one of the RENPs emission bands. Upon oxidation, the optical properties (*e.g.*, absorbance) of the FRET acceptor change and the otherwise quenched emission band from RENPs is restored. This strategy evidences that the oxidation of the FRET acceptor implies the non-reversibility of the sensing process. In this regard, the design of reversible probes for *in vivo* multivariate diagnosis and an expansive library of potentially detectable analytes represent exciting research opportunities.

Advantages associated with the lifetime-based imaging can also be translated into the field of PL thermometry, which, as a steady-state PL approach, has been a staple for over a decade with RENPs.^103^ Temperature-dependent alteration of non-radiative relaxations within specific Ln^3+^ excited states results in a change of the RENPs PL intensity and the excited state lifetime. This lifetime temperature dependence enables measuring temperature more reliably at different tissue depths and obtaining 2D thermal images, as shown by the work of Tan *et al.* (Fig. 9 – B).^102^ Hence, it has far reaching potential as a basic research tool to study the relationships between biological activity, disease, and thermal gradients with micrometric resolution *in vivo*.

As can be witnessed from the examples given, the outstanding optical properties of the Ln^3+^ series makes it possible to develop multimodal and novel imaging techniques for improved *in vivo* bioimaging with RENPs. Furthermore, their long emission lifetimes offer reliable quantification capabilities in multivariate diagnosis and nanothermometry.

## 6. Novel materials and hybrid structures

In the previous sections we have discussed the most relevant examples of NPs used for diagnostics and therapy in the NIR. However, there is a battery of new emerging materials whose PL properties can be tuned to this wavelength range^104,105^ showing prospects for future *in vivo* applications. Some of them are known and well-studied materials in other research fields, which nonetheless display intriguing characteristics at the nanometric scale and are relevant to biological studies, such as carbon or silicon NPs. Both are highly biocompatible and biodegradable, which is desirable for biomedical applications. Silicon NPs emitting in the NIR-I have been traditionally obtained by synthetic methods such as laser ablation or thermo- and pyrolysis of silicon precursors.^106^ Emission in the NIR-II can be achieved by increasing the size of the silicon NPs and even longer emission wavelengths can be accessed by doping with impurities.^107^ In addition, the lifetime and emission wavelength of these NPs can be increased by annealing at an appropriate temperature, subsequently enabling the use of time-gated imaging to remove autofluorescence of tissues in the NIR.^108^

Carbonaceous materials, such as graphene NPs, are a biocompatible, water soluble alternative to SNCs. Although their quantum confinement-defined intrinsic PL lies in the ultraviolet to visible range, recent examples have shown that this can be shifted to the NIR through the modification of the synthesis process,^109–111^ or judicious doping,^112^ both yielding materials with PL properties tuned to the NIR-I or NIR-II. This way, reduced graphene oxide-derived NPs have been added to the existing library of NIR nanothermometers.^111^ In addition, their strong absorption makes them ideal light-to-heat converters^110,112,113^ for *in vivo* PTT and PL imaging when the absorption bands are tuned to the NIR.^110^ Finally, an adequate doping can also change graphene NPs sensitivity to the environment, which has been applied for pH sensing in the NIR.^114^ It is also worth noting that other carbonaceous materials, such as single walled carbon nanotubes, can have an intrinsic PL in the NIR-II. This enabled PL imaging of the brain through intact skull with better spatial resolution than traditionally-used CT and MRI, and avoiding the need of craniotomy, cranial windows or skull-thinning required for NIR-I PL imaging.^115^

In addition to graphene, other two-dimensional materials could be used in the form of NPs with maintained or even improved optical properties at the nanoscale through their preparation by top-down synthetic procedures. Examples of these are Ti_2_N MXene NPs^116^ or Bi_2_O_2_Se NPs.^117^ Furthermore, these materials can be biodegradable, thus ensuring excretion from the body after effective PA imaging-guided PTT.^116,117^

Other major example of alternative NIR agents are perovskites. They are known for their successful application in photovoltaics and recently have attracted attention in other research fields, such as *in vivo* bioimaging and therapy.^118^ Intriguingly, the proper combination of dopant and host material can convert perovskites into persistent luminescent NPs. The emission of this kind of NPs lasts long (minutes) after the excitation is ceased, so they could be optically excited before being injected into an animal, eliminating side effects associated with the continuous irradiation of the subject and interference of the excitation light and autofluorescence in PL images. The most studied persistent PL perovskites for *in vivo* applications to date are Cr^3+^-activated phosphors emitting in the NIR-I. Additional or alternative doping with Sr^3+^ or Ln^3+^ ions could enhance the persistence of the luminescence and shift it to reach NIR-II.^119,120^ Pellerin *et al.*, showed that co-doping of LaAlO_3_:Cr^3+^ nanoperovskites with Sm^3+^ can improve their persistent radiance 35-fold after ceasing ultraviolet charging.^121^ After PEGylation, these NPs presented increased circulation time in blood vessels and could be used for NIR-I bioimaging of live mice. Although promising, persistent PL bioimaging presents some drawbacks, such as the impossibility of recharging the probes since that are excited with ultraviolet and visible light. As a first step to overcome this limitation, Hang *et al.* developed persistent Cr^3+^-activated Na_0.5_Gd_0.5_TiO_3_ perovskite-like NPs. These NPs could be excited at the lower bound of the NIR-I for *in situ* recharging with low-power incoherent light, solving the problem of fast decay that hampers the use of persistent NPs as continuous PL contrast agents.^122^ In a similar direction, a recent study demonstrated the use of RENPs as persistent luminescence NIR contrast agents upon X-ray charging.^123^

**Fig. 10:**
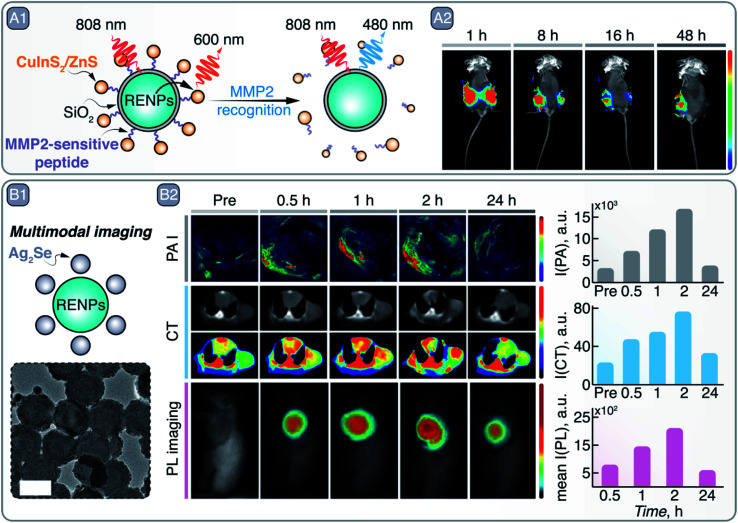
A – MMP2 recognition and tumor site imaging: A1 – schematic representation of RENPs-CuInS_2_/ZnS SNCs hybrid NPs, where upconversion PL of RENPs is alleviated from quenching by the SNCs in the presence of MMP2. A2 – time-dependent *in vivo* imaging of Cal27/VC (left side of the mouse) and Cal27/MMP2 (right side of mouse) induced tumors with RENPs-CuInS_2_/ZnS SNCs. Adapted from ref. 128 under ACS AuthorChoice License. B – Multimodal imaging with RENPs-Ag_2_S SNCs hybrid NPs: B1 – schematic representation and TEM image of *in situ* grown Ag_2_S on top of chitosan-coated RENPs. B2 – Multimodal imaging with these hybrid NPs: PA imaging (PAI), CT, and PL imaging. Absolute changes in the signal intensity over time for different imaging modalities is also quantitatively shown on the right. Adapted from ref. 130, Copyright (2020), with permission from Elsevier.

As mentioned before, the combination of different materials, such as perovskites and Ln^3+^ ^124–126^ could lead to the development of NPs with combined functionalities to overcome the limitations of the individual materials. For example, despite the outstanding properties of RENPs (see Section 5), they present low absorption. This drawback can be minimized combining them with more absorbing NPs, such as Ag_2_S SNCs, which can act as sensitizers to enhance the emission of the RENPs through an energy transfer process. This type of system has been used to detect the existence of tumors *in vivo* when the two types of NPs are crosslinked *via* glutathione, which is highly abundant in tumor microenvironments.^127^ Other examples of energy transfer-based sensors use RENP as donors to create nanocomposites in combination with SNCs for tumor detection. Chan *et al.* detected the disassembly of the RENPs-SNCs hybrid structure (NaYF_4_:Yb^3+^, Tm^3+^/NaYF_4_:Yb^3+^, Nd^3+^ RENPs and CuInS_2_/ZnS SNCs) at the tumor site in a mouse model, produced by the digestion of the link between the two PL agents by the matrix metalloproteinases (MMP2), highly expressed at the tumoral site (Fig. 10 – A1).^128^ Disassembly of the hybrid NPs decreased the SNCs emission intensity and increased that of the RENPs (Fig. 10 – A2). Similarly, two emitting NPs with different response to the environment could be combined to develop ratiometric sensors, where one of the emissions is used as a reference.^129^

Energy-transfer-based sensors can suffer from low energy transfer efficiency, low photostability, complex preparation methods and quenching of the donor emission due to the action of the acceptor, all of which should be considered when choosing the building blocks of the hybrid NPs. This latter drawback, nonetheless, can be converted into an asset for the development of a sensing platform. Shu *et al.* combined Ag_2_S SNCs and MnO_2_ nanosheets in a single nanostructure for the detection of glutathione.^131^ In the absence of the analyte, the NIR PL of the Ag_2_S SNCs was quenched by MnO_2_ nanosheets due to the overlap between the emission and absorption spectra. In the presence of glutathione, the PL of the nanoconstruct increased due to the elimination of the quencher by the target analyte (*i.e.*, MnO_2_ reduced to Mn^2+^, which changed its absorption band). This change in the emission signal was used to detect the presence of glutathione in human serum samples.

Regarding the synthetic process of hybrid systems, *in situ* growth methods present some advantages compared to other methods used to combine different NPs, such as simplicity and mild reaction conditions, and the possibility of obtaining more uniform and stable nanoplatforms. As an example, Ag_2_S SNCs can be grown *in situ* on the surface of chitosan-coated NaYF_4_:Yb^3+^, Er^3+^/NaLuF_4_:Nd^3+^, Yb^3+^/NaLuF_4_ RENPs (Fig. 10 – B1).^130^ In this way, PL and CT imaging *via* RENPs can be combined with PA imaging and PTT enacted by Ag_2_S SNCs, resulting in multimodal imaging of tumors and their destruction (Fig. 10 – B2). As another example of the combination of different NPs for the construction of multi-modal contrast agents, Zhang *et al.* used commercially available InP/ZnS SNCs to decorate the surface of silica-coated gold nanorods (GNRs).^132^ After PEGylation and modification with c(RGDfC) peptide, the dual imaging capabilities (CT, due to the GNRs, and PL imaging, due to the SNCs) of the nanocomposite was demonstrated both *in vitro* and *in vivo*. Specific recognition of HeLa and MCF-7 tumor cells was observed *via* both CT and PL imaging with the peptide-modified nanocomposite. Similarly, after injection of the nanocomposite in a nude mouse bearing a HeLa transplanted tumor, the malignant tissue started to become visible *via* both imaging approaches 6 days after injection, indicating selective nanocomposite accumulation. Another relevant example of *in situ* growth of hybrid nanoprobes is provided by H. Ning *et al.*, who developed KMnF_3_:Yb^3+^, Er^3+^/ZnS NPs that combine upconversion emission of RENPs with downshifting luminescence of SNCs.^133^ At the same time, the ZnS shell provided shielding for Ln^3+^ from external quenchers, thus boosting upconversion emission intensity.

Other strategies of hybrid NP preparation make use of cell membranes to wrap the individual entities together, which endows hybrid structures with additional functionalities and biocompatibility. Ding *et al.*, created a hybrid nanostructure formed by DNA-functionalized Ag_2_S linked to Fe_3_O_4_ magnetic NPs that were wrapped in a tumor and white blood cell hybrid membrane.^134^ These membranes increased the affinity for the cancerous cells and reduced the interaction with the white blood cells to minimize interference. High-specificity binding of the hybrid NPs to CTCs in peripheral blood samples from cancer patients was observed. The magnetic properties of the Fe_3_O_4_ NPs were used to efficiently isolate the cells using a magnetic field and the NIR emission of the Ag_2_S was used for high-sensitivity detection and quantification of CTCs. In addition, Ag_2_S SNCs and Fe_3_O_4_ magnetic NPs have also been combined by polymeric encapsulation for PL thermometry controlled magnetic hyperthermia.^135^ In this case, the temperature increase produced by the Fe_3_O_4_ NPs when subjected to an alternating magnetic field was monitored using the temperature-dependent PL of the Ag_2_S NPs. Thanks to the combined properties of their building blocks, the hybrid NPs were also exemplary multimodal contrast agents for MRI, PA and NIR-II PL imaging, as well as OCT and CT.

Finally, hybrid NPs allow the combination of multiple therapies using a single agent for a greater treatment success, such as chemotherapy and PTT.^29,136^ Alternative silica nanocapsule strategies enable combination of theranostics and multimodal imaging approaches in a straightforward manner,^137^ while classic mesoporous silica NPs can be loaded with anticancer drugs to deliver them into the tumor region. Song *et al.* designed Ag_2_S SNCs coated with mesoporous silica and grafted with graphene oxide on its surface. The mesoporous silica was also loaded with DOX, and additionally coated with FA for tumor targeting.^138^ The heat generated by Ag_2_S NPs after 808 nm irradiation was used to perform the PTT and to trigger the release of the anticancer drug by the detachment of the graphene oxide coating due to the temperature increase. The combination of both chemotherapy and PTT increased the efficacy of the treatment and only mice treated with the hybrid NPs showed no tumor recurrence.

In summary, judicious selection and combination of already established materials and novel ones in a single platform enables multimodal imaging and theranostics that could overcome the existing limitations of their individual components.

## 7. Conclusions and outlook

This review evidences that the research on novel materials capable of multimodal imaging, multivariate diagnostics, and theranostics is a highly active and promising area. This is possible thanks to the improved communication between researchers in the materials and life sciences. Yet, to translate these nanomaterials from preclinical research to the clinics will necessitate further, extensive collaboration between multiple disciplines. At this stage, the translation of these cutting-edge materials to the clinics remains out of reach and more work needs to be done to demonstrate their viability in the clinical settings.

Usual arguments to interest clinicians in these optical materials (*e.g.* the lack of ionizing radiation) are insufficient. This is exacerbated by the availability of true and tested traditional technologies (such as CT or MRI). Unlike these traditional technologies, however, new nanomaterials provide qualitative as well as quantitative information about physiological parameters of relevance, such as temperature or presence of chemical species in the environment that indicate disease. Further, nanomaterials can provide a therapeutic stimulus concurrent to diagnosis, resulting in targeted disease therapy with reduced side effects. In this review, we show there is substantial evidence for the potential and effectiveness of multivariate diagnostics (imaging and sensing) and theranostics (imaging and therapy) by NIR-active NPs and nanostructures. It is time to share these results with the clinicians, a significant number of whom will be undoubtedly surprised by the copious research on nanomaterials and will be interested in their use for imaging and sensing. Notwithstanding, the bio and medical communities are increasingly studying the interaction of the NPs with cells, tissues, and biological models; specifically, how the type, state, or physiology of the living organism can influence the performance of the NPs.^139^

Nonetheless, even for the most interested clinicians, two looming questions prevail: “are these nanomaterials toxic?”; and “how are they cleared from the body following administration and operation?”. Here, our answers are not so convincing. Results included in this review reveal that materials science researchers are also worried about these questions, but are more focused on the improvement of nanomaterials synthesis procedures and on the design of *in vivo* proof-of-concept experiments to demonstrate their potential in biomedical setting. While almost every manuscript on nanomaterials intended for biological applications includes basic toxicity and biodistribution analyses, some important aspects are often avoided. This includes the integrity of NPs, particularly hybrid ones, after administration, the stability of NPs in biological media and at physiological temperatures, particularly for extended periods of time, and the effects of long-term accumulation. Thus, it is not surprising that the available evidence is not enough to convince physicians. In this respect, the scientific community working on nanomaterials for biomedicine should clearly define the standards of toxicity, safety, and clearance of their materials and the best practices to assess these parameters. It is mandatory to define and identify few but concise experiments (*in vitro* and *in vivo*) that would be considered as golden rules to elucidate how the organism reacts to the administration of NIR-emitting NPs. Despite recent attempts to standardize reporting in bio-nano experimental literature, there has been little uptake of these practices by the materials science community.^140,141^

Furthermore, materials are continuously being improved and pushed forward in consecutive steps, which demonstrates an amazing body of innovation and interest in this research area. Nonetheless, must every iteration of each nanomaterial be tested *in vivo*? An ethical argument should also be made to reduce animal testing for the sake of routine proof-of-concept demonstrations, which often bring little scientific value if any at all. It is our believe and hope that more humane technologies, such as 3D cell cultures, lab grown tissues, and organs-on-a-chip, will become staple approaches to investigate nanomaterials at primary and pre-clinical stages of their development.

Standardization is also lacking regarding the optical properties of most of the nanomaterials discussed in this review. Development and improvement of nanomaterials is an ever-on-going process and synthesis technologies that ensure good batch-to-batch reproducibility and high-quality NPs are not always straightforward to create. As a representative example, Ag_2_S SNCs are not yet considered robust clinical probes because of the lack of reproducibility in their optical properties. These depend strongly on both the synthesis and the storage conditions, resulting in a large inter- and intra-batch variability. This is also the case for some of the RENPs discussed in this review. Again, the key point here is the standardization, together with the publication of works with meticulous description of synthesis procedures. This is, without a doubt, a required step towards commercialization of NIR-emitting NPs for biomedical applications. Notwithstanding, the development of cost-effective and green synthesis procedures is highly desirable.

It has been observed along this review that adding the possibility of sensing to imaging makes NPs especially suitable for advanced diagnostics. However, this is an emerging field and sensors' susceptibility to cross-talk (*i.e.* similar responses to changes in different environmental parameters) is hampering their development. To address this problem, new systems with sensitivity to a single parameter exclusively are in demand. This requires a complete knowledge of the mechanisms governing the light–matter interactions in these nanostructures so that the response to different external stimuli can be decoupled. Here, standardization is once again crucial. Further development of the field of NP-based biosensing requires the widespread acceptance by the scientific community of standards for the characterization of sensing units. Such standards will focus on the elucidation of possible bias and, in addition, will describe in detail the measuring conditions for a characterization to be accepted by the scientific community.

Despite the required work in standardizing synthesis methods, characterization techniques, and toxicity and biocompatibility assays, the works highlighted in this review showcase the fast development of NPs intended for biomedical use. This is the best evidence we have for the interest of a broad scientific community in the development of multifunctional NPs for multivariate diagnostics. This review also evidences the great potential of NIR-emitting NPs for early diagnosis and efficient treatment of different diseases, and highlights the high degree of multidisciplinary efforts necessary to spark the interest of a broader scientific community towards a unified goal. It is time to think even harder from the materials science standpoint about how to develop the required standards while designing nanomaterials with carefully tailored properties to meet a specific purpose.

## Conflicts of interest

The authors declare that there are no conflicts of interest.

## Supplementary Material
